# Methods of induction of labor and women’s experience: a population-based cohort study with mediation analyses

**DOI:** 10.1186/s12884-021-04076-x

**Published:** 2021-09-14

**Authors:** Pauline Blanc-Petitjean, Corinne Dupont, Bruno Carbonne, Marina Salomé, François Goffinet, Camille Le Ray, for the MEDIP study group, Catherine Crenn-Hebert, Adrien Gaudineau, Frédérique Perrotte, Pierre Raynal, Elodie Clouqueur, Gaël Beucher, Catherine Deneux-Tharaux, Pierre-Yves Ancel

**Affiliations:** 1 Université de Paris, CRESS, INSERM, INRA, F-75004 Paris, France; 2 Department of Obstetrics and Gynecology, AP-HP, Louis Mourier Hospital, DHU Risks in pregnancy, Université de Paris, F-92700 Colombes, France; 3 Réseau périnatal Aurore - Hôpital de la Croix Rousse, Université Lyon 1, HESPER EA 7425 Health Services and Performance Research, F-69008 Lyon, France; 4 Department of Obstetrics and Gynecology, Princess Grace Hospital, Monaco, France; 5 AP-HP, URC-CIC Paris Descartes Necker/Cochin, F-75014 Paris, France; 6 AP-HP, Cochin Hospital, Port Royal Maternity Unit, DHU Risks in Pregnancy, Université de Paris, F-75014 Paris, France

**Keywords:** Induction of labor, Cervical ripening, Maternal experience, midwifery research, causal mediation analysis

## Abstract

**Background:**

Negative childbirth experience may affect mother wellbeing and health. However, it is rarely evaluated in studies comparing methods of induction of labor (IoL).

**Aim:**

To compare women’s experience of IoL according to the method, considering the mediating role of interventions and complications of delivery.

**Methods:**

We used data from the MEDIP prospective population-based cohort, including all women with IoL during one month in seven French perinatal networks. The experience of IoL, assessed at 2 months postpartum, was first compared between cervical ripening and oxytocin, and secondarily between different cervical ripening methods. Mediation analyses were used to measure the direct and indirect effects of cervical ripening on maternal experience, through delivery with interventions or complications.

**Findings:**

The response rate was 47.8% (*n* = 1453/3042). Compared with oxytocin (*n* = 541), cervical ripening (*n* = 910) was associated less often with feelings that labor went ‘as expected’ (adjusted risk ratio for the direct effect 0.78, 95%CI [0.70–0.88]), length of labor was ‘acceptable’ (0.76[0.71–0.82]), ‘vaginal discomfort’ was absent (0.77[0.69–0.85]) and with lower global satisfaction (0.90[0.84–0.96]). Interventions and complications mediated between 6 and 35% of the total effect of cervical ripening on maternal experience. Compared to the dinoprostone insert, maternal experience was not significantly different with the other prostaglandins. The balloon catheter was associated with less pain.

**Discussion:**

Cervical ripening was associated with a less positive experience of childbirth, whatever the method, only partly explained by interventions and complications of delivery.

**Conclusion:**

Counselling and support of women requiring cervical ripening might be enhanced to improve the experience of IoL.

**Supplementary Information:**

The online version contains supplementary material available at 10.1186/s12884-021-04076-x.

## Tweetable abstract

In the French current practice, women’s experience of induction of labor was less positive with cervical ripening, whatever the method, compared to oxytocin infusion: results of the MEDIP population-based cohort of women with induction of labor.

## Statement of significance

### Problem

Experience of induction of labor (IoL) is rarely assessed according to the method and results found in RCT are not extrapolable in current practice.

### What is already known

Negative experience of IoL can have short- and long-term effects on mother well-being and health and may be explained both by the methods and the outcomes of IoL.

### What this paper adds

Using population-based data, experience of Iol was less positive with cervical ripening. Experience of IoL was not different according to the ripening method and was only partly explained by the interventions and complications.

## Introduction

Women’s experience and satisfaction with childbirth is an important element for judging the quality of care in a maternity ward [1]. From 5 to 20% of women describe their experience of delivery as negative [2–4]. These negative experiences may have short- and long-term effects: impairment of the mother-child bond from the very start, postpartum depression, decisions to not become pregnant again, fear of delivery, and requests for a repeat cesarean for a subsequent pregnancy [5–9]. Generally, patients’ experience in hospitals is best when the medical outcomes of an intervention are both good and uncomplicated [10]. Women’s experience in childbirth is known to be worse when they have a cesarean delivery, especially when it is performed as an emergency [11, 12].

One of the most common interventions in obstetrics today is induction of labor (IoL). In most developed countries, one woman in five has labor induced [13–15]. The choice of the method depends in part on the clinical examination of cervical ripeness. Intravenous oxytocin infusion and amniotomy are recommended when the cervix is favorable, otherwise cervical ripening is necessary to increase the likelihood of successful induction [16]. Several methods of cervical ripening are commonly used because the data currently available do not justify to recommend one method over any other [17–22].

Most studies comparing methods of IoL have assessed their effectiveness and safety. Following a Delphi process, Dos Santos et al. [23] listed maternal experience of childbirth and satisfaction in the set of core outcomes that should routinely been assessed in studies dealing with IoL. A review of the literature has shown that only around 5% of the trials of induction report women’s experience of it [24]. The extrapolation of the results of trials is questionable because the populations included are highly selected and the women receive both standardized management and special attention because of their participation in the trial [25]. To our knowledge, no observational study has assessed women’s experience according to the method of induction in routine care with population-based data.

The objective of this study was to compare, in a population-based cohort of women who underwent induction of labor, the experience of delivery according to the method used, taking into account the mediating contribution of intervention and complication of delivery.

## Material and methods

This was a comparative study using the data of the MEDIP (Methods of induction of labor and perinatal outcomes) prospective population-based cohort. The objective of the MEDIP study was to describe the practices of induction in France and to compare the effectiveness and safety of the different methods in current use [19]. This assessment of women’s experience was one of the planned secondary objectives of the study.

The cohort included all women who had labor induced from 17 November to 21 December 2015 in all maternity units belonging to 7 perinatal health networks (*n* = 94). These units accounted for one-sixth of the annual deliveries in France and were representative of the entire set of French maternity units [26]. Still birth and medically-indicated termination of pregnancy were exclusion criteria. A follow-up of two-months was performed.

Data were recorded prospectively. The midwife or obstetrician managing the woman informed the indication of IoL, the method used and the Bishop score at the onset of induction. The choice of method was based on each maternity ward’s habits or was left to the practitioner’s choice. Independent clinical research technicians recorded additional data from the woman’s medical records. Experience of induction was assessed with a self-administered questionnaire sent by mail or e-mail (three reminders) at 2 months postpartum.

**Fig. 1:**
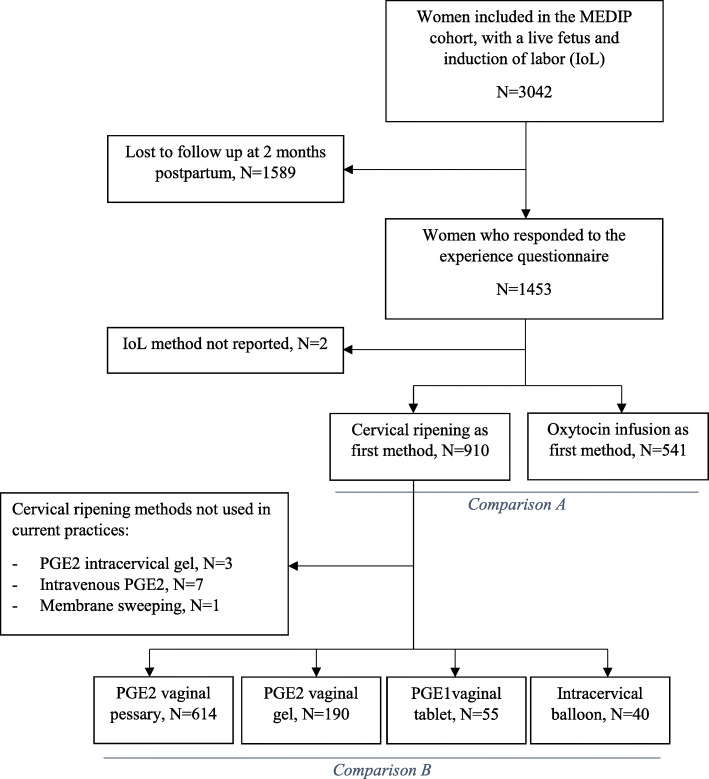
Flowchart of study population. Legend: Iol, induction of labor; PG, prostaglandins

The experience of IoL was first compared between women who underwent cervical ripening as first method of IoL and those who did not, i.e. those who received oxytocin infusion as first method (exposure/outcomes associations A). Secondarily, the experience of delivery was compared according to each method of cervical ripening (exposure/outcomes associations B). We compared the methods mainly used in the MEDIP cohort: vaginal dinoprostone pessary as a slow-release insert (as reference, because it was the method used most often), dinoprostone as a vaginal gel, misoprostol as a vaginal tablet and an intracervical balloon (Cook® balloon or Foley catheter). We excluded women who received PGE2 intracervical gel (*n* = 3) or intravenous PGE2 (*n* = 7) or membranes sweeping only (*n* = 1) because the number of women was too small to perform statistical comparisons for these methods (Fig. 1). In the MEDIP study, all methods of labor induction were used individually, with no combination of two methods simultaneously. Specific modes of usage of the methods in this cohort have previously been described [27].

For assessing women’s experience, a specific questionnaire was developed after a review of qualitative research on the topic by the study Scientific Committee, a multidisciplinary group of perinatal professionals, epidemiologists and user representatives [28–30]. We analysed eight self-administered questions about the course of labor and delivery, vaginal discomfort, maximum pain, global satisfaction and the likelihood that woman would choose the same method of induction again. Some categories were regrouped to obtain binary outcomes to study positive experience of childbirth (Additional file 1).

A directed acyclic graph (http://www.dagitty.net/) presented the assumed exposure-outcome pathway with confounding and mediators variables (Additional file 2). We considered that the outcomes of induction were mediators in the causal pathway between the induction method used and the experience of induction. For this reason, we did not adjust for these variables [31]. To take them into account, the outcomes of IoL were integrated as mediators. Mediation analysis decompose the total effect of the causal pathway between an exposure and an outcome into a direct and an indirect effect. The indirect effect estimated is the extent to which the method of labor induction affects women’s experience through the outcomes of delivery. The outcomes of induction were summarized in a composite variable called ‘delivery with intervention or complication’, that is, any combination of a cesarean or operative vaginal delivery, postpartum hemorrhage (total estimated blood loss ≥500 ml), severe perineal laceration or NICU admission. We adjusted for parity and history of C-section, body mass index, peridural analgesia, maternal age, medical indication and maternity status. We also adjusted for Bishop score when measuring the association between the experience of delivery according to each method of cervical ripening (B) but not for the first comparisons (A). In fact, adjusting for Bishop score may lead to overfitting because it is strongly correlated with the choice of performing cervical ripening or not. Interaction for parity and medical indication of IoL was tested.

### Statistical analyses

Multivariable Poisson regression models were performed to obtain risk ratios. A robust variance was used to take the cluster effect into account. For the mediation analysis, we used the inverse odds weighting (IOW) method described by Nguyen et al. [32, 33] IOW is a weight-based approach that condenses the association between the exposure and the mediator into a weight. It is a stepwise process. First, we estimated for each woman the predictive odds of ‘undergoing cervical ripening’ due to a ‘delivery with intervention or complications’. The inverse of this predicted odds gives a weight. Secondarily, the direct effect of cervical ripening on maternal experience was estimated by using weighted Poisson regression. Then, the indirect effect was calculated by subtracting the direct from the total effect coefficients. The proportion mediated (PM) was calculated as the ratio of the total effect to the indirect effect [34]. Confidence intervals for indirect effect and PMs were obtained by bootstrapping. Complete case analyses were performed because outcome data were missing for fewer than 2% of the women who responded to the questionnaire. The statistical analyses were performed with Stata/SE software, version 15.0.

## Results

Of the 3042 women included in the MEDIP cohort, 1453 (47.8%) responded to the self-administered questionnaire at 2 months postpartum. (49% by mail and 51% by e-mail). The respondents included a higher proportion of women who were nulliparous, older than 35 years, born in Europe, living with a partner, who belonged to higher socio-professional categories and who did not receive intracervical balloon for IoL. The response was not associated with more unfavorable outcomes or with medical interventions during delivery (Fig. 1).

Women’s characteristics of respondents and experience according to method of induction are described in Table 1 and Table 2. Compared with women receiving oxytocin (*n* = 541), those who underwent cervical ripening (*n* = 910) had a less positive experience of induction. After taking the confounding and mediating factors into account, cervical ripening was significantly associated with a lower risk of experiencing that ‘labor went quite normally’ (RR adjusted for direct effect, aRR 0.86, 95% CI 0.81–0.93), that ‘labor and delivery went as expected’ (direct aRR = 0.78, 95% CI 0.70–0.88 and aRR 0.88, 95% CI 0.79–0.98) and that the ‘length of labor was acceptable’ (direct aRR 0.76, 95% 0.71–0.82). Cervical ripening was also associated with less ‘absence of sensation of vaginal discomfort’ (direct aRR 0.77, 95% CI 0.69–0.85), maximum pain ‘less than 8/10’ (direct aRR 0.59, 95% CI 0.51–0.70), a poorer ‘global satisfaction’ (direct aRR 0.90, 95% CI 0.84–0.96), and less willingness to have another induction ‘by the same method’ (direct aRR 0.83, 95% CI 0.78–0.88). Tests of interaction for parity and medical indication for induction of labor were non-significant. Between 6 and 35% of the association between cervical ripening and experience was significantly mediated by the variable ‘delivery with intervention or complications’ (Table 3).

**Table 1:** Characteristics of study population according to the first method of labor induction

Characteristic of study population	Cervical ripening *N* = 910	Oxytocin and/or amniotomy *N* = 541
**Age, years**	30.9 (4.9)	31.8 (4.8)
**Country of birth**		
Europe	639 (81.9)	364 (81.8)
North Africa	75 (9.6)	48 (10.8)
Sub-Saharan Africa	30 (3.9)	15 (3.4)
Other	36 (4.6)	18 (4.0)
**Maternal occupation**		
Higher professional occupation	213 (26.4)	129 (27.1)
Intermediate occupation	278 (34.5)	161 (33.8)
Sales and service worker	184 (22.8)	102 (21.5)
Skilled or unskilled manual workers	18 (2.2)	11 (2.3)
Unemployed or not in the labor force	113 (14.0)	73 (15.3)
**BMI before pregnancy, kg/m**^**2**^	24,8 (5.6)	23.9 (4.6)
**Nulliparous**	592 (65.4)	185 (34.3)
Parous, no previous CS	297 (32.8)	216 (60.1)
Parous, previous CS	16 (1.8)	30 (5.6)
Antenatal education with information about IoL	160 (29.6)	298 (32.9)
**Bishop score**	3 [2–4]	5 [5–7]
**Non-medical induction**	43 (4.7)	100 (18.5)
**Gestational age, WG**	40 [38–41]	40 [38–41]
**Epidural analgesia**	773 (85.0)	486 (89.8)
**Mode of delivery**		
Spontaneous vaginal	537 (59.3)	417 (77.2)
Instrumental vaginal	147 (16.2)	56 (10.4)
Cesarean	222 (24.5)	67 (12.4)
**Time to delivery < 24 h**	568 (63.9)	535 (99.8)
**Episiotomy**^**a**^	191 (28.0)	66 (14.0)
**Severe perineal laceration**^**a**^	8 (0.9)	3 (0.6)
**PPH**	54 (6.0)	32 (5.9)
**NICU hospitalisation**	58 (6.4)	40 (7.4)
**Birth with intervention or complication**^b^	516 (57.9)	214 (40.2)

Data are expressed as n (%), mean (sd) or median [25th–75th percentile]; BMI, body mass index; CS, cesarean section; IoL, induction of labor, PPH, postpartum hemorrhage; NICU, neonatal intensive care unit

^a^For women with vaginal delivery

^b^Composite variable: cesarean or operative vaginal delivery, episiotomy, severe perineal laceration, PPH or NICU hospitalisation

**Table 2:** Experience of labor induction according to the method used (cervical ripening versus oxytocin and/or amniotomy)

Experience of labor induction	Cervical ripening ***N*** = 910 N(%)	Oxytocin and/or amniotomy ***N*** = 541 N(%)
Labor went quite normally	558 (62.1)	438 (81.3)
Labor proceeded just about as expected	405 (44.9)	352 (65.4)
Length of labor was acceptable	530 (59.2)	444 (83.0)
Delivery proceeded exactly as expected	413 (45.9)	341 (63.6)
Absence of vaginal discomfort during the induction	450 (50.2)	364 (67.7)
Maximum pain perceived, numeric scale < 8/10	252 (28.8)	247 (47.1)
Globally satisfied about the induction	626 (69.6)	445 (83.0)
If labor had to be induced again, the same method would be liked	615 (58.9)	432 (86.5)

**Table 3:** Multivariable association between cervical ripening and maternal experience, mediated by interventions or complications of delivery

Experience of labor induction	Cervical ripening versus oxytocin and/or amniotomy (Reference)			
	Direct effect aRR (95% CI)	Indirect effect aRR (95% CI)	Total effect aRR (95% CI)	% mediated^**a**^ (95% CI)
Labor went quite normally	0.86 (0.81, 0.93)	0.96 (0.95, 0.98)	0.83 (0.77, 0.89)	21.4% (8.5, 34.2)
Labor proceeded just about as expected	0.78 (0.70, 0.88)	0.96 (0.94, 0.98)	0.75 (0.67, 0.84)	14.8% (4.6, 25.0)
Length of labor was acceptable	0.76 (0.71, 0.82)	0.98 (0.96, 0.99)	0.74 (0.69, 0.80)	7.7% (1.4, 13.9)
Delivery proceeded exactly as expected	0.88 (0.79, 0.98)	0.93 (0.91, 0.96)	0.82 (0.74, 0.92)	34.6% (5.2, 64.0)
Absence of vaginal discomfort during the induction	0.77 (0.69, 0.85)	0.98 (0.96, 1.00)	0.75 (0.68, 0.83)	6.4% (−1.6, 14.3)
Maximum pain perceived, numeric scale < 8/10	0.59 (0.51, 0.70)	1.04 (1.00, 1.08)	0.62 (0.53, 0.72)	−8.9% (−17.4, −0.3)
Globally satisfied about the induction	0.90 (0.84, 0.96)	0.97 (0.96, 0.99)	0.87 (0.82, 0.93)	21.1% (0.6, 41.5)
If labor had to be induced again, the same method would be liked	0.83 (0.78, 0.88)	0.98 (0.97, 0.99)	0.82 (0.77, 0.87)	8.5% (0.6, 16.4)

^a^Estimation of the size of the indirect effect mediated by delivery with intervention/complication (i.e. cesarean, operative vaginal delivery, postpartum hemorrhage, severe perineal laceration or neonatal intensive care unit hospitalisation): ((βtotal-βdirect)/βtotal)*100; All models adjusted for: parity, previous cesarean, body mass index, maternal age, medical indication for induction, maternity unit status and epidural analgesia

Comparing the dinoprostone insert (reference) to the other types of prostaglandins, the experience of IoL did not differ significantly for most items, except that more women who received the intravaginal misoprostol tablet did not experience vaginal discomfort (direct aRR 1.34, 95% 1.20–1.48) (Table 4). The intracervical balloon was associated with more frequent rating of maximum pain lower than 8/10 (direct aRR 1.78, 95% CI 1.20–2.65).

**Table 4:** Experience of induction of labor according to the different methods of cervical ripening

Experience of labor induction	Dinoprostone pessary ***N*** = 614		Dinoprostone gel ***N*** = 190		Misoprostol tablet ***N*** = 55		Intracervical balloon ***N*** = 40	
	n (%)	aRR (95% CI) ^a^	n (%)	aRR (95% CI) ^a^	n (%)	aRR (95% CI) a	n (%)	aRR (95% CI) a
Labor went quite normally	370 (61.0)	1.00 (Reference)	117 (62.9)	1.02 (0.87, 1.18)	35 (63.6)	1.04 (0.87, 1.24)	28 (71.8)	1.03 (0.81, 1.31)
Labor proceeded just about as expected	261 (42.8)	1.00 (Reference)	87 (46.8)	1.31 (0.84, 1.37)	27 (49.1)	1.14 (0.91, 1.44)	22 (55.0)	1.25 (0.86, 1.83)
Length of labor was acceptable	359 (59.1)	1.00 (Reference)	102 (55.7)	0.93 (0.78, 1.10)	36 (65.5)	1.09 (0.93, 1.28)	26 (65.0)	1.07 (0.77, 1.49)
Delivery proceeded exactly as expected	265 (43.7)	1.00 (Reference)	90 (48.4)	1.07 (0.88, 1.29)	26 (47.3)	1.03 (0.78, 1.35)	26 (65.0)	1.38 (1.00, 1.35)
Absence of vaginal discomfort during the induction	297 (49.0)	1.00 (Reference)	92 (49.2)	0.97 (0.82, 1.13)	37 (67.3)	1.34 (1.20, 1.48)	18 (47.4)	1.23 (0.89, 1.70)
Maximum pain perceived, numeric scale < 8/10	170 (32.3)	1.00 (Reference)	57 (34.6)	1.03 (0.82, 1.29)	19 (35.9)	1.31 (0.96, 1.80)	21 (56.8)	1.78 (1.20, 2.65)
Globally satisfied about the induction	425 (69.7)	1.00 (Reference)	130 (70.7)	1.00 (0.90, 1.12)	37 (67.3)	0.98 (0.87, 1.10)	27 (67.5)	0.87 (0.70, 1.08)
If labor had to be induced again, the same method would be liked	398 (65.9)	1.00 (Reference)	139 (75.5)	1.11 (0.98; 1.26)	42 (77.8)	1.18 (0.96; 1.46)	27 (67.5)	0.90 (0.70; 1.16)

^a^Estimation of the direct effect after taking mediation of delivery with intervention/complication into account. All models adjusted for: parity, previous cesarean and Bishop score

## Discussion

### Principal findings

Experience of induction of labor was less positive for women requiring cervical ripening. Women deplored a greater gap between what was expected and what was experienced, more unacceptable duration of labor, vaginal discomfort, intense pain, and insatisfaction with induction. Most of the experience was explained by the method and not by the interventions or complications of delivery. The women’s experience did not seem different between the prostaglandins but ripening with the balloon catheter seemed associated with less intense pain.

### Strengths and limitations

This is to our knowledge the first study to examine specifically multiple domains of the experience of IoL associated with cervical ripening with population-based data while taking into account the mediating role of interventions and complications. The MEDIP study was specifically designed to study perinatal outcomes associated with the different methods of induction of labor in France [19, 26]. The prospective data collection about the course of induction ensured the quality of the information reported.

Our study had some limits. A non-validated questionnaire was used, because the existing scales did not appear appropriate for a specific evaluation of the experience of IoL. Since the MEDIP study was performed, the EXIT scale (Experience of induction tool) has been developed in Australia for this purpose, but it has never been validated in another population [35]. External validity is also questionable because the women included came from perinatal networks not randomly selected, although characteristics of maternity units participating in this large sample were comparable to those of all French maternity units [26]. There was also a selection bias of respondent women who were most frequently European and employed. This questionnaire was sent at 2 months because memories of facts immediately after delivery may be modified, due to tiredness or lack of time to integrate the course of events. Moreover, interviewing women during their stay in the maternity unit may have led the woman interviewed to provide information that she felt comfortable with the care providers. We cannot know whether the responses were biased toward women with less or worse positive experience or not. Nonetheless, we showed that the response was not associated with interventions or complications. Finally, a strong proportion of the experience was probably affected by the environment of birth, the specific organization of a maternity ward or the individual relation created between the woman and the provider supporting and caring for her [36, 37]. Such information might have been particularly relevant for comparison of different cervical ripening methods, for which the usage are heterogeneous and depend on maternity unit’s preferences [26].

### Interpretation

These data about how women experience IoL according to the method used might be relevant for guiding the management of care. Qualitative studies have already shown that a negative experience of induction was associated with a lack of preparation and information about the benefits and risks of induction and its course, to the intensity of pain, the duration of the induction and to a poorer medical outcome, in particular, emergency cesarean delivery [36, 37]. These studies didn’t distinguished specifically women with cervical ripening. When comparing woman satisfaction between oxytocin alone and vaginal prostaglandins E2, a meta-analysis of Alfirevic et al. [16] previously found no difference. However, the three trials included had a different way of measuring maternal satisfaction and the studies were probably not representative of actual obstetric practice.

The less positive experience of childbirth with cervical ripening does not imply that oxytocin should be proposed whatever the degree of cervix immaturity. Indeed, cervical ripening is recommended in case of unfavourable cervix [38, 39]. However, it calls for stronger support and counselling. In France, most pregnant women attend antenatal birth classes, especially the nulliparous women, but the content of these classes are probably very heterogeneous. Antenatal education on what to expect after induction on labor according to the method should probably be enhanced [13]. Moreover, one-to-one support for management of pain, which is not usual in French practices would certainly be good for improving maternal experience [37].

Our results also raise questions, as the number of elective IoL might be increased in the coming years. Indeed, recent data have demonstrated better perinatal outcomes with elective induction of labor at term, regardless of cervical status [40–42]. In the ARRIVE trial comparing induction of labor with expectant management among 6000 low-risk nulliparous women at 39 weeks of gestation, induction of labor resulted in a better global experience and less pain [43]. Results were not stratified according to the method of induction. One limitation is that the experience of women who agreed to participate to such trial may not be representative of that of women in general population. They volunteered to participate and may have been more inclined to experience their induction positively whatever the method used. In the review of observational study comparing induction of labor with expectant management, women’s experience was not evaluated [42]. Our results can not imply that women who had unfavourable cervix expectantly managed instead of being induced would have had a better experience. However, in the absence of a medical indication, waiting for the cervix to be more favourable may be an alternative to improve maternal experience.

The few studies comparing the experience of different methods of cervical ripening also found greater discomfort and stronger pain during induction by prostaglandins compared with the balloon, but overall global satisfaction did not differ [44, 45]. This may be explained by the fact that the balloon appears to have a mechanical ripening action but not contraction-inducing effect that may be less painful. These results merit further exploration in view of the small number of women induced with the balloon included in these studies as in our study.

## Conclusion

In current practice, cervical ripening was associated with a less positive experience of Iol, the major part of it was not explained by the interventions and complications of delivery. Counselling and support of women requiring cervical ripening might improve the experience of induction of labor. Further data about experience according to the method of cervical ripening are necessary.

## Supplementary Information

**Additional file 1: Table.** Items of the self-administered questionnaire evaluating the positive experience of induction of labor.

**Additional file 2: Figure.** Directed acyclic graph for the association between the method of labor induction and maternal experience. Legend: DAG created with the web-based application DAGitty (http://www.dagitty.net/). CS: caesarean section, BMI: body mass index.

**Additional file 3: Table.** Characteristics and major outcomes of women who did and did not respond to the questionnaire.

## Data Availability

The datasets used and/or analyzed during the current study are available from the corresponding author on reasonable request.

## Abbreviations

Iol: Induction of labor
BMI: Body mass index
CS: Cesarean section;
PPH: Postpartum hemorrhage
NICU: Neonatal intensive care unit
RCT: Randomized controlled trial
