# Supplementary material for: Methods of induction of labor and women’s experience: a population-based cohort study with mediation analyses
Source: BMC Pregnancy Childbirth. 2021 Sep 14;21:621. doi: 10.1186/s12884-021-04076-x (PMC8442398; doi:10.1186/s12884-021-04076-x)
Supplement: Supplementary file 1 — Additional file 1: Table. Items of the self-administered questionnaire evaluating the positive experience of induction of labor. [file 12884_2021_4076_MOESM1_ESM.docx]

**Additional file 1: Items of the self-administered questionnaire evaluating the positive experience of induction of labor**

| **Questionnaire items** | **Potential responses** | **Categories defining positive experience of IoL** |
| --- | --- | --- |
| 1. Would you say that your labor went quite normally? | 1 (absolutely agree), 2 (agree), 3 (more or less agree), 4 (neither yes or no), 5 (more or less disagree), 6 (disagree), 7 (completely disagree) | From 1 to 3 |
| 1. Would you say that your labor proceeded just about as you had expected? | 1 (absolutely agree) to 7 (completely disagree) | From 1 to 3 |
| 1. Would you say that the length of your labor was acceptable? | 1 (absolutely agree) to 7 (completely disagree) | From 1 to 3 |
| 1. Would you say that your delivery proceeded exactly as you had expected? | 1 (absolutely agree) to 7 (completely disagree) | From 1 to 3 |
| 1. Did you feel any vaginal discomfort during induction? | 0 (No), 1 (Yes, but the discomfort was tolerable), 2 (Yes, and the discomfort was unbearable) | 0 |
| 1. What is the maximum pain you felt? | 0 (no pain) to 10 (unbearable pain) | Less than 8  (median of the distribution) |
| 1. Overall, regarding your labor and delivery, would you say that you are: | 1 (very satisfied), 2 (somewhat satisfied), 3 (moderately satisfied), 4 (somewhat dissatisfied), 5 (very dissatisfied) | 1 and 2 |
| 1. If you were to have labor induced again during a future pregnancy, would you like the same method to be used? | 0 (No), 1 (Yes) | 1 |

IoL: induction of labor
