# Supplementary figures and images for: Methods of induction of labor and women’s experience: a population-based cohort study with mediation analyses
Source: BMC Pregnancy Childbirth. 2021 Sep 14;21:621. doi: 10.1186/s12884-021-04076-x (PMC8442398; doi:10.1186/s12884-021-04076-x)

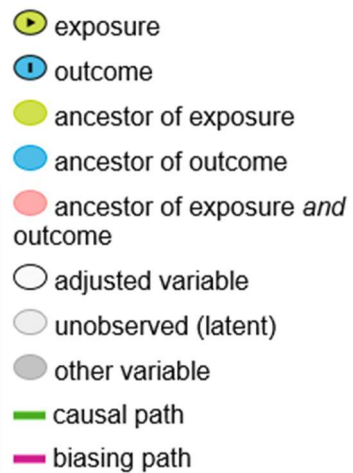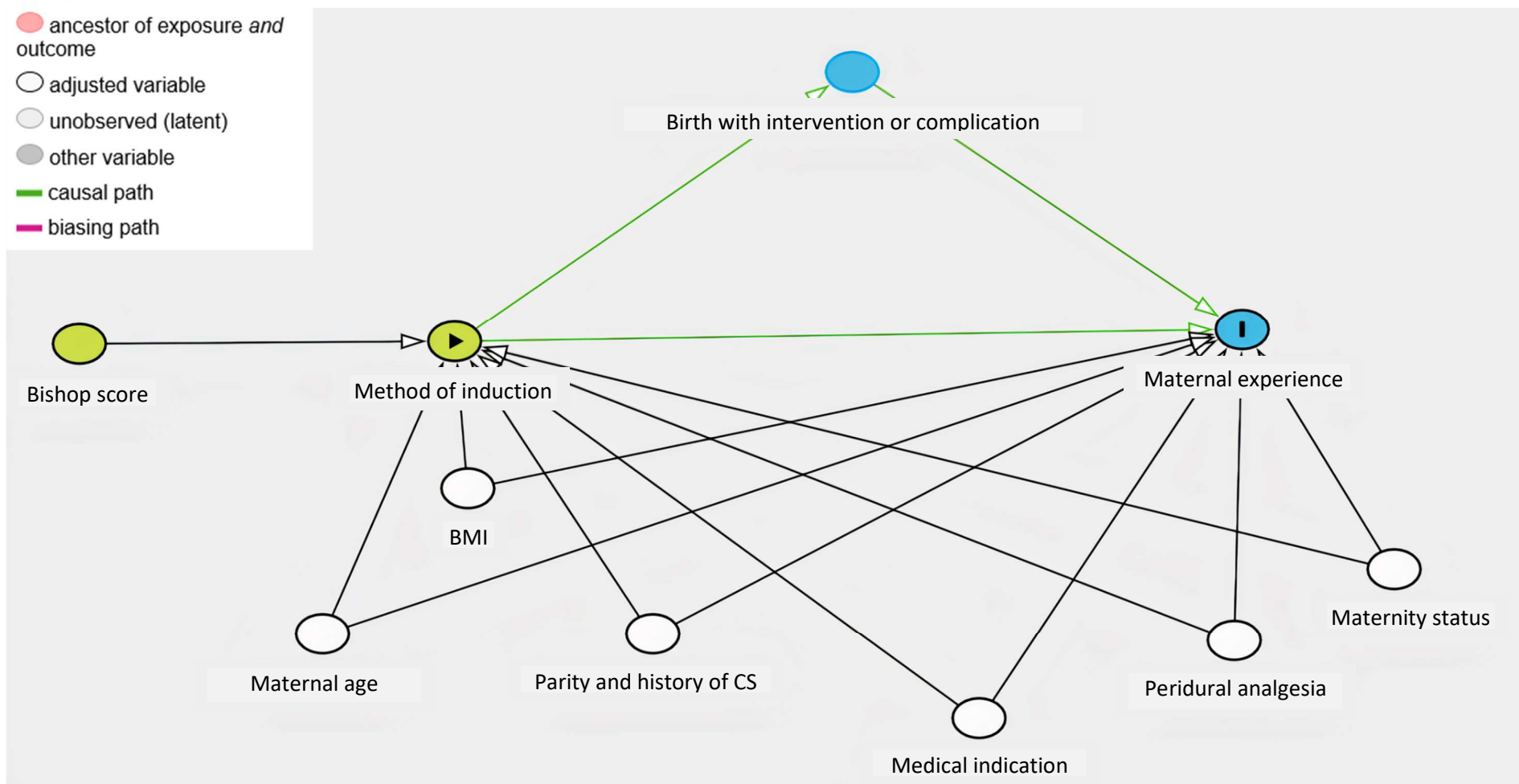

Supplement: Supplementary file 2 — Additional file 2: Figure. Directed acyclic graph for the association between the method of labor induction and maternal experience. Legend: DAG created with the web-based application DAGitty (http://www.dagitty.net/). CS: caesarean section, BMI: body mass index. [file 12884_2021_4076_MOESM2_ESM.pdf]
