# Supplementary material for: Methods of induction of labor and women’s experience: a population-based cohort study with mediation analyses
Source: BMC Pregnancy Childbirth. 2021 Sep 14;21:621. doi: 10.1186/s12884-021-04076-x (PMC8442398; doi:10.1186/s12884-021-04076-x)
Supplement: Supplementary file 3 — Additional file 3: Table. Characteristics and major outcomes of women who did and did not respond to the questionnaire. [file 12884_2021_4076_MOESM3_ESM.docx]

**Additional file 3: Characteristics and major outcomes of women who did and did not respond to the questionnaire**

| **Characteristics** | **MEDIP population**  **N=3042** | **Respondents**  **N (%)**  **n=1453** | **Non-respondents**  **N (%)**  **n=1589** | ***P*** |
| --- | --- | --- | --- | --- |
| **Age, years, mean (SD)** | 30.8 (5.3) | 31.2 (1.9) | 30.5 (±5.5) | <0.001 |
| **Geographic origin, n(%)** |  |  |  | <0.001 |
| Europe | 1836 (72.5) | 1003 (81.9) | 833 (63.7) |  |
| North Africa | 412 (16.3) | 123 (10.0) | 289 (22.1) |  |
| Sub-Saharan Africa | 144 (5.7) | 45 (3.7) | 99 (7.6) |  |
| Other | 140 (5.5) | 54 (4.4) | 86 (6.6) |  |
| **BMI before pregnancy, kg/m^2^** | 24.7 (5.4) | 24.5 (5.2) | 24.9 (±5.2) | 0.02 |
| **Nulliparous, n (%)** | 1495 (49.5) | 777 (53.8) | 718 (45.5) |  |
| **Maternal occupation** |  |  |  | <0.001 |
| Higher professional occupation | 548 (20.7) | 342(26.7) | 206 (15.1) |  |
| Intermediate occupation | 786 (29.7) | 439 (34.2) | 347 (25.4) |  |
| Sales and service workers | 601 (22.7) | 286 (22.3) | 315 (23.1) |  |
| Skilled or unskilled manual workers | 84 (3.2) | 29 (2.3) | 55 (4.0) |  |
| Unemployed or not in labor force | 631 (23.8) | 186 (14.5) | 445 (32.5) |  |
| **Status of maternity unit** |  |  |  | 0.26 |
| University public | 921 (30.3) | 415 (28.6) | 506 (31.8) |  |
| Other public | 1071 (35.2) | 520 (35.8) | 551 (34.7) |  |
| Private | 706 (23.2) | 518 (34.7) | 552 (33.5) |  |
| **Medical indication** | 2736 (90.2) | 1037 (90.1) | 1429 (90.2) | 0.94 |
| **Gestational age, WG** |  |  |  |  |
| **Cervical ripening** | 1880 (61.9) | 910 (62.7) | 970 (61.2) | 0.40 |
| **Method of cervical ripening** |  |  |  | <0.001 |
| PGE2 vaginal pessary | 1245 (66.2) | 614 (67.5) | 631 (65.1) |  |
| PGE2 vaginal gel | 359 (19.1) | 190 (20.9) | 169 (17.4) |  |
| PGE1 vaginal tablet | 117 (6.2) | 55 (6.0) | 62 (6.4) |  |
| Intracervical balloon | 138 (7.3) | 40 (4.4) | 98 (10.1) |  |
| Other | 21 (1.1) | 11 (1.2) | 10 (1.0) |  |
| **Epidural analgesia** | 2652 (87.2) | 1259 (86.7) | 1393 (87.7) | 0.40 |
| *(To continue)* |  |  |  |  |
| **Mode of delivery** |  |  |  | 0.30 |
| Spontaneous vaginal | 1958 (64.7) | 954 (66.0) | 1004 (63.5) |  |
| Instrumental vaginal | 429 (14.2) | 203 (14.0) | 226 (14.3) |  |
| Cesarean | 639 (21.1) | 289 (20.0) | 350 (22.2) |  |
| **Birth with intervention or complication*** | 1533 (51.2) | 730 (51.2) | 803 (51.2) | 0.98 |

BMI: body mass index, WG: weeks of gestation; ^*^Cesarean or operative vaginal delivery, episiotomy, severe perineal laceration, PPH, neonatal NICU transfer
